# A meta‐analysis of the relation between hippocampal volume and memory ability in typically developing children and adolescents

**DOI:** 10.1002/hipo.23414

**Published:** 2022-03-17

**Authors:** Morgan Botdorf, Kelsey L. Canada, Tracy Riggins

**Affiliations:** ^1^ Department of Psychology University of Maryland College Park Maryland USA; ^2^ Institute of Gerontology Wayne State University Detroit Michigan USA; ^3^ Present address: Department of Psychology University of Pennsylvania Philadelphia Pennsylvania USA

**Keywords:** adolescent, child, hippocampus, memory, meta‐analysis

## Abstract

Memory is supported by a network of brain regions, with the hippocampus serving a critical role in this cognitive process. Previous meta‐analyses on the association between hippocampal structure and memory have largely focused on adults. Multiple studies have since suggested that hippocampal volume is related to memory performance in children and adolescents; however, the strength and direction of this relation varies across reports, and thus, remains unclear. To further understand this brain–behavior relation, we conducted a meta‐analysis to investigate the association between hippocampal volume (assessed as total volume) and memory during typical development. Across 25 studies and 61 memory outcomes with 1357 participants, results showed a small, but significant, positive association between total hippocampal volume and memory performance. Estimates of the variability across studies in the relation between total volume and memory were not explained by differences in memory task type (delayed vs. immediate; relational vs. nonrelational), participant age range, or the method of normalization of hippocampal volumes. Overall, findings suggest that larger total hippocampal volume relates to better memory performance in children and adolescents and that this relation is similar across the memory types and age ranges assessed. To facilitate enhanced generalization across studies in the future, we discuss considerations for the field moving forward.

## 1 INTRODUCTION

Memory is a critical component of everyday life and is important for an array of outcomes. Across development, this cognitive process helps children learn about the world and form memories of events (Keresztes et al., 2018; Mullally & Maguire, 2014; Shing et al., 2010). The brain plays a role in supporting memory throughout childhood and adolescence with regions like the hippocampus, prefrontal cortex, and posterior parietal cortex serving key roles in memory processing (Ghetti & Bunge, 2012; Ofen, 2012; Riggins et al., 2020; Shing et al., 2016). Although this larger network of brain regions is important for the development of memory abilities, work in both animal and human samples has continued to demonstrate the critical role of the hippocampus in the formation and consolidation of memories (Davachi et al., 2003; Eichenbaum, 2004; Lavenex & Banta Lavenex, 2013; Scoville & Milner, 1957). Volumetric differences in the structure of hippocampus relate to memory ability in adult and child samples (e.g., DeMaster et al., 2014; Lee et al., 2020; Riggins et al., 2018). However, the strength and direction of this association varies across studies, especially in child and adolescent samples. Given these mixed findings and the importance of understanding the neural correlates of memory during development, there is great value to clarifying this brain–behavior association in developmental samples using a meta‐analytic approach.

### 1.1 Structural development of the hippocampus

The hippocampus continues to mature throughout childhood and adolescence, a finding documented in vivo through assessing changes in volume over time (e.g., Gogtay et al., 2006). Recent longitudinal studies report that postnatal development of the hippocampus is characterized by a slight positive increase in total volume throughout childhood and adolescence (Canada et al., 2020; Tamnes et al., 2018). Given its heterogeneous nature, the hippocampus can be divided into subunits, including subfields and subregions, both of which exhibit differential developmental trajectories (Lavenex & Banta Lavenex, 2013; Poppenk et al., 2013). Subfields, including cornu ammonis (CA) regions 1–4, dentate gyrus (DG), and subiculum, are distributed along the longitudinal axis of the hippocampus and are functionally and structurally distinct subunits of the hippocampus (Lavenex & Banta Lavenex, 2013; Seress, 2001). Subregions, including head/body/tail or anterior (head)/posterior (body and tail) divisions, are divided along the longitudinal axis of hippocampus and exhibit differential structural and functional connectivity with other neural structures (Poppenk et al., 2013).

### 1.2 Development of memory

Paralleling neural changes in the hippocampus during childhood are age‐related improvements in memory ability, which vary with memory type. For example, substantial gains are often seen on tasks assessing relational memory, a type of memory that requires the binding of features, between 4 and 7 years of age (also referred to as associative memory, contextual memory, or relational binding; Olson & Newcombe, 2014; Riggins, 2014). Relational memory continues to develop well into adolescence while nonrelational memory, which does not require binding of information, is relatively mature by early childhood (Lee et al., 2020). Although there may be age‐related gains in nonrelational memory performance, these are less dramatic in comparison to those in relational memory tasks (Riggins, 2014; Sluzenski et al., 2006).

In addition to differences in memory related to binding, the length of time memories can be retained improves during development. Children are better able to retain memories over a short delay earlier in childhood, with the ability to retain memories over longer delays increasing across development (Saragosa‐Harris et al., 2021). These various forms of memory not only develop at different rates but are thought to differentially rely on the hippocampus. Functional MRI studies in adults and children show that the hippocampus exhibits a greater neural response for memory processing that requires binding of elements (i.e., relational memory) than for memory processing that does not include such binding (Davachi & Wagner, 2002; Ghetti et al., 2010). In addition, rodent and human studies highlight the critical role of hippocampus in the consolidation of long‐term memories (Cohen & Eichenbaum, 1993; Eichenbaum & Cohen, 2001). Thus, it is important to consider the differential role the hippocampus plays in different types of memory (relational vs. nonrelational, immediate vs. delayed) when investigating brain–behavior associations.

### 1.3 Associations between hippocampal volume and memory

Results of studies assessing hippocampal volume and memory in children and adolescents vary greatly. These studies range from documenting a significant, positive association between memory and hippocampal volume (e.g., Bauer et al., 2019; Cooper et al., 2015; Lambert et al., 2019), to a negative association (e.g., Schlichting et al., 2017; Willoughby et al., 2008), and even no association or differential associations when assessing subregion or subfield volumes (e.g., Daugherty et al., 2017; DeMaster et al., 2017; Riggins et al., 2015).

The mixed nature of these findings in developmental samples is similar to that seen in studies focused on adult samples, which have reported both a significant association between total hippocampal volume and memory (e.g., Hardcastle et al., 2020) and no association (e.g., Clark et al., 2020) or only in those with spatial expertise (i.e., London taxi drivers; Woollett & Maguire, 2011). A useful approach to gain a clearer picture of the association between the hippocampus is meta‐analysis. A previous meta‐analysis examining the relation between the hippocampus and memory from middle childhood through adulthood suggested that smaller hippocampal volume relates to better memory in school‐aged children and adolescents (ages 7–17 years old) and poorer memory in older adults (>50 years, Van Petten, 2004). However, conclusions from the developmental sample were limited as only two studies included children and adolescents, due to the limited work in developmental populations at the time.

### 1.4 Factors that may obscure hippocampal volume memory associations

Over the last 10 years, studies have accumulated examining the association between hippocampal volume and memory in children and adolescents. However, it remains difficult to extrapolate this relation given that studies and their results vary greatly on several factors, including: (1) the type of memory task used, (2) the age of participants, and (3) the method used to normalize hippocampal volumes (e.g., none vs. covariance).

First, the type of memory task varies widely across studies. This is an important factor to consider given that the hippocampus is thought to be critical for consolidation of memory, especially relational memory (Eichenbaum & Cohen, 2001). Therefore, tasks that assess relational memory and delayed memory, which allow consolidation to take place, likely rely on the hippocampus more than nonrelational memory or immediate/short delay memory tasks (though the hippocampus' role in relational binding mechanisms has been observed over short intervals as well; Olson & Newcombe, 2014). Although the hippocampus plays a key role in relational and delayed memory, developmental research has used a variety of lab‐based memory assessments. Studies have used immediate memory tasks, which typically require the retrieval of information within 10 min of encoding (e.g., Children's Memory Scale [CMS] Immediate Story Recall; NIH Toolbox Picture Sequence Memory Task; Test of Memory and Learning [TOMAL] Immediate Recall), and delayed memory tasks, which typically require the retrieval of information after >10 min from encoding (e.g., Source memory; CMS Delayed Story Recall; Rey Complex Figure Task [RCFT] Delayed Recall). Studies have also used nonrelational memory tasks (e.g., CMS Story Recall; TOMAL Recall; RCFT Delayed Recall), which do not require that representations are bound together, and relational memory tasks (e.g., Associative Inference; Contextual Memory; Paired Associates Learning; Relational Memory Task; Source Memory; Triplet Binding Task), which require that representations are bound together.

Differences in the age of participants may also result in discrepant findings. Studies show that there are differential associations with subregions and subfields, in particular, in different age groups (e.g., DeMaster et al., 2014; Riggins et al., 2018; Schlichting et al., 2017). For example, Schlichting et al. (2017) showed differential associations between hippocampal head volume and CA1 volume and inference performance among younger (~6‐year‐old), middle (~17‐year‐old), and older individuals (~29‐year‐old). Given that memory continues to improve throughout childhood and adolescence, paralleled by continued maturation of specific subfields of hippocampus, observed brain–behavior relations may differ across developmental periods (Lavenex & Banta Lavenex, 2013; Lee et al., 2016). Some studies include participants across a large age range, spanning both childhood and adolescence (and sometimes adulthood, e.g., Daugherty et al., 2017; Horner et al., 2012; Østby et al., 2012), whereas other studies focus on a smaller age range (e.g., 8–11 years, DeMaster et al., 2014 or 4 and 6 years, Riggins et al., 2015). By combining age groups, associations that are present at one point in development (but not another) may be masked, contributing to variations in findings.

Finally, the approach used to account for overall brain volume may contribute to variations in results. When including hippocampal volumes in analyses, best practices suggest that differences in brain volume (i.e., total brain volume [TBV] or intracranial volume [ICV]) should be considered; however, there is little agreement as to how this should be accomplished. Some studies include TBV or ICV as a covariate in analyses. Other studies adjust hippocampal volumes to account for TBV or ICV using an analysis of covariance approach (Raz et al., 2005) and may apply different adjustments to different groups based on age and/or sex (Keresztes et al., 2017). Still other studies divide hippocampal volumes by TBV or ICV (O'Brien et al., 2011) or use raw hippocampal volumes. Although research shows limited effects of normalization method on results in adult samples (Van Petten, 2004), it may account for more variability in findings from developmental populations where relatively more neural changes are occurring in addition to physical growth.

Mixed results from developmental studies examining the relation between hippocampal volume and memory are unfortunate as a clear understanding of this brain–behavior association is vital for understanding both typical and atypical development. The hippocampus is known to be a stress‐sensitive brain region and is impacted by various disorders (McLaughlin et al., 2019; Woon & Hedges, 2008). Research has started to link structural variations in the hippocampus with various forms of psychopathology given deficits in memory abilities that are often observed in disorders, such as posttraumatic stress disorder and depression (e.g., Kribakaran et al., 2020; Postel et al., 2019). For example, adolescents with depressive symptoms exhibit differences in hippocampal volumes compared to those who do not show symptoms (Redlich et al., 2018). These differences in hippocampal structure likely have implications for cognitive and behavioral processes that rely on hippocampus. Given these associations with risk for psychopathology, clarifying typical associations will allow for a better understanding of the impact of atypical hippocampal development on cognition, including memory and other processes reliant on the hippocampus.

### 1.5 The current study

In the current study, we used a meta‐analytic approach to synthesize findings across studies and determine the strength and direction of the relation between total hippocampal volume and memory in childhood and adolescence. Using a meta‐analytic technique allowed us to move beyond the limited conclusions drawn from variable results of single studies by combining estimates across studies. Furthermore, in this study we explored the extent to which this brain–behavior relation differed depending on (1) the memory task used (i.e., immediate vs. delayed recall; nonrelational vs. relational memory), (2) the age range of participants, and (3) the method used to normalize volumes. Another variation in the current literature is whether the hippocampus is assessed as a homogenous (i.e., total hippocampal volume) or heterogeneous structure (i.e., hippocampal subfield/subregion volumes). The limited number of studies that assessed subregion volumes and memory (10 studies) and subfield volumes and memory (8 studies) prevented a thorough meta‐analysis; however, we include a preliminary qualitative discussion of the literature examining subfield and subregion volumes in relation to children's memory ability.

We hypothesized a positive relation between total hippocampal volume and memory given research showing that the developmental trajectory of the hippocampus in childhood and adolescence is characterized by small gains in total volume (Canada et al., 2020; Tamnes et al., 2018). However, given the immense developmental changes occurring in the brain from childhood to adolescence, it is possible that associations may differ by age. We also hypothesized a stronger association between hippocampal volume and memory for tasks that assessed relational or delayed memory based on literature in adults that suggests this type of specialization (e.g., Eichenbaum & Cohen, 2001; Olson & Newcombe, 2014).

## 2 METHODS

### 2.1 Inclusion criteria

Studies included in the current meta‐analysis met the following criteria: included participants between 2 and 18 years old, assessed associations between hippocampal volume and memory, and focused on typically developing participants.

### 2.2 Literature search

To complete a thorough search of the literature, we used relevant search terms including Boolean terms: “children or youth or adolescents or teenagers or young adults or students or preschool‐age or school‐age” AND “memory or episodic memory or binding or long‐term memory or relational binding or associative memory” AND “hippocampus or hippocampal or hippocampal volume or hippocampal structure or hippocampal subregions or hippocampal subfields or hippocampal development.” We searched various directories including APA PsycInfo, Education Resources Information Center, Family and Society Studies Worldwide, open dissertations, EBSCO Psychology and behavioral sciences collection, and PubMed. The literature search was completed in May 2020. This search returned 7967 results (after deduplication, 5004 unique articles were identified for screening, see below).

In addition to searching the literature, we reached out to individual researchers who may have relevant unpublished work and posted a call for unpublished data on listservs. This was an a priori attempt to address possible publication bias given that the published literature can be a biased survey of the landscape. Two unpublished studies were included in the meta‐analysis.

### 2.3 Abstract screening

Following the literature search, article citations were saved in Zotero and deduplicated to ensure that each article was only included once. Five thousand four citations remained after deduplication. Two screeners (M.B. and K.L.C) completed abstract screening in Abstrakr beta using hierarchical questioning (http://abstrackr.cebm.brown.edu). A total of 114 studies remained following abstract screening.

### 2.4 Coding the literature

Coding was then completed to record statistics for each study along with other relevant study information (e.g., demographic information, memory task information). Coding was completed by M.B., K.L.C., and a third trained person. The focus was on bivariate correlations between hippocampal volume and memory tasks as this statistic allowed for comparing findings across multiple studies. Studies differed in how they reported test statistics. When bivariate correlations were not provided or were stated as nonsignificant (without a statistic reported), we contacted the authors. When the authors responded, we included the updated value. If we did not receive a response, the partial correlation was included, if available. If the partial correlation was not available, the study was not included in analyses. This resulted in the exclusion of four studies. When correlations were reported separately by hemisphere (i.e., left/right hemisphere), the average correlation was calculated (*n* = 5 studies) to maximize the amount of data used and minimize the number of analyses.

The meta‐analytic approach used in the current study did not require the tasks to be independent if they are from the sample study (Hedges et al., 2010). A strength of this approach is that the selection of a single outcome is not imposed by the researcher as it allows for dependence of measures and for multiple measures of memory to be included. Therefore, if a study included multiple dependent memory variables, data was recorded for all variables given that the tasks assessed declarative memory processes, or the study authors referred to the task as assessing memory. For two studies in which the number of variables exceeded five, the five variables that best exemplified memory were chosen to limit the influence of a single study in analyses.

The definition of delayed memory varied across studies with some including a 5‐min delay and others including a 1‐h or 1‐week delay. For this meta‐analysis, tasks with >10 min for recall were classified as delayed recall tasks and tasks with <10 min for recall were classified as immediate memory tasks (see Table S1). This cut‐off is supported by work in developmental samples which has shown that recall after a 10‐min delay is similar to recall after a 48‐h delay (Bauer et al., 1999). Furthermore, a review of the rodent literature showed that hippocampal lesions impaired memory after a delay of 10 min or greater (Cohen & Stackman, 2015). Tasks that included a component where there was binding of two or more elements were coded as relational memory tasks. Only tasks with a clear binding component were labeled as such (see Table S1). Tasks that primarily assessed attention or executive functions were not included.

Age range was coded as one of four categories (early childhood, middle childhood, adolescence, and mixed range). Study age ranges were coded as early childhood if they included participants younger than 8 years old and middle childhood if they included participants between 8 and 12 years old. Study age ranges were coded as adolescence if they included participants between 12 and 18 years old and mixed range if the age range of participants spanned three or more of these developmental periods (e.g., 6–17 years old). If an age range was not large, yet still spanned two developmental periods, it was included with the period that had the majority of the ages in it (e.g., 7–11 years old). Six studies were classified as early childhood, six studies as middle childhood, six studies as adolescence, and seven studies as mixed range for the meta‐analysis assessing total hippocampal volume.

For longitudinal studies, only the first time point of data was included. For clinical studies, results were included from the control group, whenever possible. If data was not reported separately for the control group, we contacted the authors. If we received a response, the updated statistic was included. If we did not receive a response from the authors, the data was not included, as we were interested in assessing typical development.

During the coding process, 89 additional studies were excluded. These studies were mainly excluded because they did not utilize MRI, did not include a memory assessment, the children were too old (i.e., mean age >18 years), or the study did not report statistics on a typically developing control group. In total, 25 studies, 61 memory measures, and 1357 participants were labeled as relevant for the meta‐analysis with total hippocampal volume. Table 1 includes all studies and relevant variables for the meta‐analysis, and Table S1 lists the memory task classification (i.e., relational, nonrelational, immediate, delayed) for each measure included in the meta‐analysis.

**TABLE 1:** Studies included in the meta‐analysis assessing total hippocampal volume and memory

Study	*N*	Mean age (years)	Age range (years)	% Female	Normalization method	Memory assessment
Barch et al. (2019)	85	15.60	13–19	53	None	NIH Toolbox Picture Sequence Memory^a^
Bauer et al. (2019)	66	7.34	5–8	49	Adjusted using ANCOVA (ICV)	Self‐Derivation through Integration (Stem Facts–Open Ended)
						Self‐Derivation through Integration (Stem Facts–Total)
						Self‐Derivation through Integration (Integration Facts–Open Ended)
						Self‐Derivation through Integration (Integration Facts–Total)
Brunnemann et al. (2013)^b^ ^,^ ^c^	19	9.00	7–11	42	Adjusted using covariance method	RCFT (Delayed Recall)^a^
Chaddock et al. (2010)	49	10.00	9–10	59	None	Item Memory (d′)
						Relational Memory Task (d′)
Cooper et al. (2015)	40	12.17	8–15	40	Adjusted using regression (ICV)	CMS Verbal/Visual (Immediate Recall)^c^
						CMS Verbal/Visual (Delayed Recall)^a^
						Memory Component^a^
DeMaster et al. (2014)	35	9.65	8–11	54	Adjusted using ANCOVA (ICV)	Color/Spatial Memory (Source Memory Index)
Dougherty and Riggins (2013)	53	7.28	5–10	49	Adjusted using ANCOVA (ICV)	CMS Stories (Immediate Recall)^a^
						CMS Stories (Delayed Recall)^a^
						Source Memory
Dudek et al. (2014)	17	12.30	11–14	41	Adjusted using ICV/Hippocampus proportion	CMS Stories (Immediate Recall)^a^
						CMS Stories (Delayed Recall)^a^
						RCFT (Delayed Recall)^a^
						TOMAL Visual Selective Reminding (Delayed)^a^
						TOMAL Word Selective Reminding (Delayed)^a^
Fuentes et al. (2012)	26	16.40	11–20	81	Adjusted using scaling factor (head/skull size)	TOMAL Word Selective Reminding (Immediate)^a^
						TOMAL Word Selective Reminding (Delayed)^a^
						TOMAL Memory for Stories (Immediate Recall)^a^
						TOMAL Memory for Stories (Delayed Recall)^a^
						TOMAL Facial Memory^a^
Hill et al. (2004)^c^	10	10.00	7–14	60	None	WRAML (Immediate Recall)^a^
						WRAML (Delayed Recall)^a^
Horner et al. (2012)	14	18.60	11–35	57	Adjusted for ICV	Source Memory
						Item Memory
Isaacs et al. (2003)^c^	8	13.67	NR	63	Adjusted for ICV	WMS Stories (Immediate Recall)^a^
						WMS Stories (Delayed Recall)^a^
						Paired Associates Learning (Immediate)
						Paired Associates Learning (Delayed)
Jabès et al. (2015)^c^	28	9.75	10	54	None	Continuous Memory Recognition Task (d′)
Lambert et al. (2017)	34	14.01	8–19	50	ICV included as covariate	Context Memory Accuracy
Lambert et al. (2019)	33	14.07	8–19	49	ICV included as covariate	Paired Associates Learning
Lambert et al. (2020)	26	13.98	9–19	NR	ICV included as covariate	Context Tasks (d′)
Lee et al. (2020)	171	9.45	7–12	49	Adjusted using ANCOVA	Triplet Binding Task (Item‐Time)
						Triplet Binding Task (Item‐Space)
						Triplet Binding Task (Item–Item)
Martinos et al. (2012)	11	2.47	NR	49	Volumes divided by ICV	Novelty Preference (Immediate)
						Novelty Preference (Delayed)
Østby et al. (2012)^b^	107	13.90	8–19	49	Adjusted for TBV	RCFT (30‐min Recall)^a^
						RCFT (1‐week Recall)^a^
						RCFT (1‐week Retention)^a^
Piccolo et al. (2018)	143	16.49	12–20	46	None	NIH Toolbox Picture Sequence Memory
Raffington et al. (2019)	82	7.19	6–7	46	None	Item‐Association Memory Task (Immediate Recall)
Riggins et al. (2015)	44	5.52	4 and 6	64	Adjusted using ANCOVA (ICV)	Source Memory
Riggins et al. (2018)	177	6.29	4–8	52	Adjusted using ANCOVA (ICV)	Source Memory
						CMS Stories (Immediate Recall)^a^
						CMS Stories (Delayed Recall)^a^
						Temporal Order Recall
Schlichting et al. (2017)	41	11.97	6–17	51	Adjusted using ANCOVA (ICV)	Associative Inference (Inference Performance)
		11.97				Associative Inference (Direct Pair Performance)
		12.00		49		Statistical Learning
Trontel et al. (2013)	31	11.98	5–19	0	ICV included as covariate	TOMAL Object Memory (Immediate Recall)^a^
						TOMAL Visual Search (Immediate Recall)^a^
						TOMAL Facial Memory (Immediate Recall)^a^
						TOMAL Visual Selective Reminding (Delayed)^a^
						TOMAL Facial Memory (Delayed Recall)^a^
Willoughby et al. (2008)	18	12.39	9–14	65	ICV/Hippocampus/proportion	CMS Stories (Immediate Recall)^a^
						CMS Stories (Delayed Recall)^a^
						CMS Word Pairs (Immediate Recall)^a^
						CMS Word Pairs (Delayed Recall)^a^
						RCFT (Delayed Recall)^a^
Yu et al. (2018)	31	10.49	8–12	42	Adjusted using ANCOVA (ICV)	Visual Auditory Learning (Immediate)
	30					Visual Auditory Learning (Delay)
Yurgelun‐Todd et al. (2003)	37	14.60	12–17	65	Volumes divided by ICV	WAIS Digit Symbol (Delayed Recall)^a^

Abbreviations: ANCOVA, analysis of covariance; CMS, Children's Memory Scale; CVLT, California Verbal Learning Test; ICV, intracranial volume; NR, not reported; RCFT, Rey Complex Figure Test; TOMAL, Test of Memory and Learning; WAIS, Weschler Adult Intelligence Test; WMS, Weschler Memory Scale; WRAML, Wide Range Assessment of Memory and Learning.

^a^ Indicates age‐adjusted memory variable.

^b^ Indicates studies that provided partial correlations (controlled for variables other than ICV or TBV).

^c^ Indicates studies that were excluded from analyses due to missing statistics.

In addition to collecting data from studies assessing total hippocampal volume, we also collected data from those that assessed subregion (i.e., anterior [head], posterior [body/tail]) or subfield volumes (i.e., CA1, DG, subiculum) in relation to memory performance. Because of the limited research, we did not run a quantitative analysis using data from these studies. Instead, we provide a preliminary qualitative overview of the subregion and subfield data in the Discussion. Our search returned 10 studies that assessed hippocampal subregion volumes and memory using 582 participants and 8 studies that assessed hippocampal subfield volume using 648 participants. Tables S2 and S3 list the relevant variables for the studies assessing hippocampal subregion and subfield volumes, respectively.

### 2.5 Statistical analysis

#### 2.5.1 Synthesizing effect sizes across studies

R Studio version 4.0.2 was used for data analysis. To complete the meta‐analysis, we used the metafor (Viechtbauer, 2010) and robumeta packages (Fisher et al., 2017). A meta‐analysis was run to assess the association between hippocampal volume and memory performance. An estimated overall correlation coefficient was calculated using a random effects model with robust variance estimation (RVE; Hedges et al., 2010), which allowed for the inclusion of dependent estimates (i.e., multiple memory outcomes for each study). Specifically, the identifier for each study was entered in the analysis, making it a random effect analysis. This method included a small sample correction to account for differences in sample size. Another option would have been to average across outcomes for each study rather than include multiple measures from the same study. Although this would have removed dependency of estimates, it would have resulted in the loss of valuable information. Nevertheless, we ran analyses averaging across tasks for each study and obtained similar results. Therefore, we report results from the analyses using RVE.

#### 2.5.2 Quantifying and explaining heterogeneity

To quantify variability in study estimates, *I* ^2^ was used, which calculates the percentage of heterogeneity that represents actual differences among studies rather than expected differences (i.e., differences that may be the result of different samples). *I* ^2^ is calculated as part of the robumeta RVE procedure (Fisher et al., 2017). *I* ^2^ less than 50% is typically thought to represent negligent or small heterogeneity whereas an *I* ^2^ larger than 50% represents moderate heterogeneity and greater than 75% represents substantial heterogeneity (Higgins et al., 2019; Huedo‐Medina et al., 2006).

To identify variables that account for heterogeneity, we focused on the those highlighted in the introduction (i.e., type of memory task, age range of participants, normalization method). Meta‐regression was used to investigate these variables that may contribute to heterogeneity in study estimates (Borenstein et al., 2009). Given the two delineations of task type, main and interactive effects between delay and relational memory were examined. Specifically, we assessed whether associations with hippocampal volume were more robust for specific task types (i.e., delay/relational, delay/nonrelational, no delay/relational, no delay/nonrelational).

#### 2.5.3 Addressing publication bias

We aimed to determine if our meta‐analytic model was robust to publication bias by (1) visually inspecting a funnel plot of the data and (2) using the trim and fill method. As an initial method to assess evidence for publication bias, a funnel plot of the data was visually examined for asymmetry. The funnel plot assumes there is an even distribution of positive and negative effects and compares the correlation coefficient to the standard error. The trim and fill method, a nonparametric method from Duval and Tweedie (2000), was used to better understand what hypothetical studies may be missing due to publication bias and how that may have affected overall estimates. This is done by iteratively removing studies with small sample sizes that may be causing asymmetry in the funnel plot and then re‐estimating the overall correlation coefficient. The trimmed studies are then added back to the plot along with imputed “missing” studies and the variance around the overall correlation coefficient is then re‐estimated. The trim and fill method was used in addition to the funnel plot as it is a less subjective way to assess publication bias compared to visually assessing the funnel plot. To complete the publication bias analysis, the agg function was used in the MAd toolbox (Del Re & Hoyt, 2014), which allowed for aggregating correlational measurements using the method by Borenstein (2009).

## 3 RESULTS

### 3.1 Total hippocampal volume and memory meta‐analyses

**FIGURE 1:**
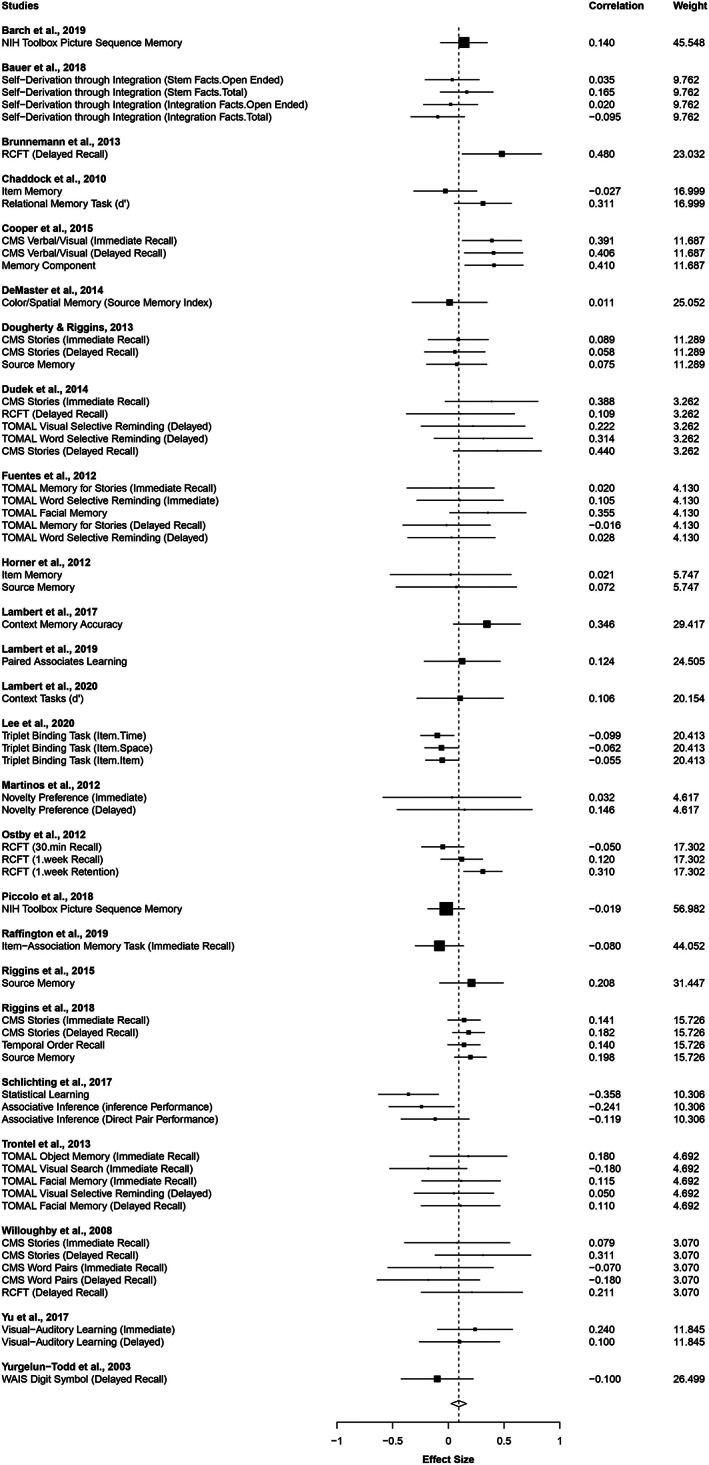
Forest plot showing the correlation coefficient and the small sample weighting correction for each study included in the meta‐analysis assessing total hippocampal volume and memory with a 95% confidence interval. Size of square for each study indicates sample size weighted by the number of measurements included in the meta‐analysis

Results showed a small, but significant, positive association between total hippocampal volume and memory performance across all tasks (*k* = 25 studies, 61 memory outcomes, overall correlation = .094, *SE* = 0.033, 95% confidence interval [CI]: [0.025, 0.163], *p* = .01; Figure 1). Correlations ranged from −.36 to .48.

The heterogeneity analysis showed that there was a small degree of heterogeneity (*I* ^2^ = 37.06%). Results of the meta‐regression showed no significant relation between task type and variability in estimates for delay (*b* = 0.063, *SE* = 0.080, *p* = .446) or relational memory (*b* = −0.068, *SE* = 0.066, *p* = .316). Furthermore, there were no significant interactive effects between delay and relational memory predicting variability in study estimates (*b* = −0.086, *SE* = 0.114, *p* = .466). Results also suggested that differences in age range (all *p*s > .05), and normalization of hippocampal volumes (*b* = −0.006, *SE* = 0.094, *p* = .951) across studies did not contribute to heterogeneity in results. Follow‐up analyses showed that publication year (*b* = 0.009, *SE* = 0.012, *p* = .504), the inclusion of partial correlations (*b* = 0.105, *SE* = 0.086, *p* = .267), sample size (*b* = −0.001, *SE* = 0.001, *p* = .416), the male/female makeup of participants (*b* = 0.0004, *SE* = 0.003, *p* = .889), and scanner type (i.e., 1.5 vs. 3 T; *b* = −0.063, *SE* = 0.068, *p* = .384) did not predict variability in study estimates.

### 3.2 Publication bias analysis

**FIGURE 2:**
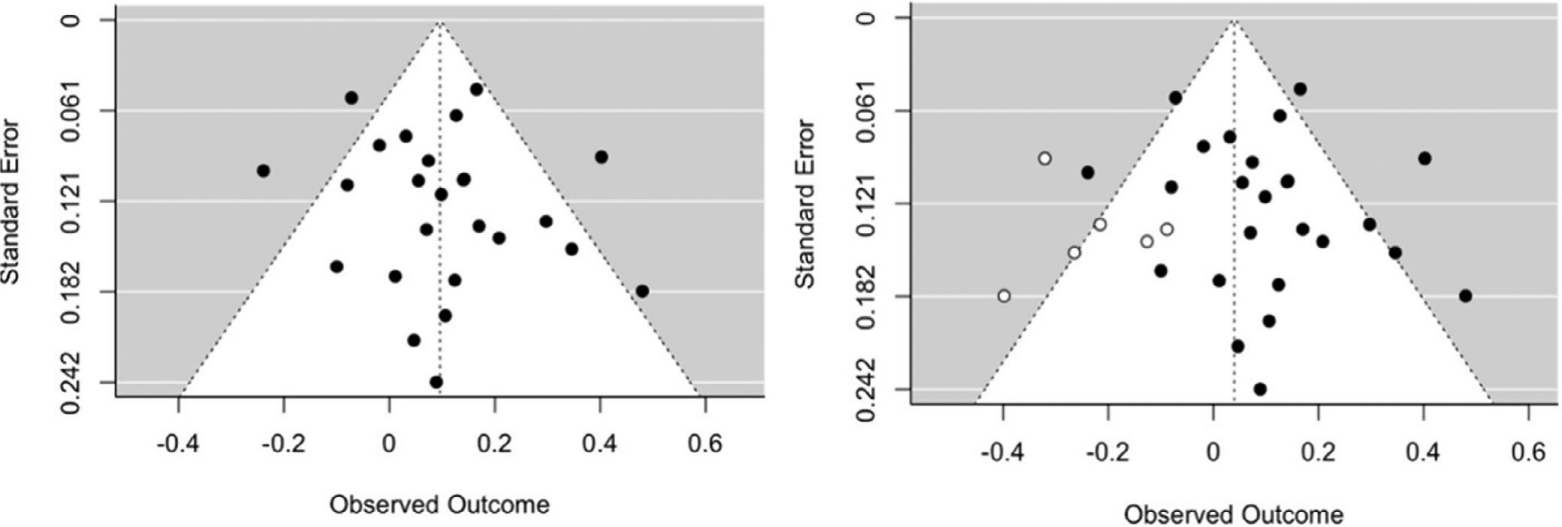
Funnel plots used to assess publication bias for studies included in meta‐analysis on total hippocampal volume and memory before (left panel) and after trim and fill analysis (right panel). Filled points represent studies included in the meta‐analysis. Unfilled points represent “missing” studies imputed from the trim and fill analysis

A funnel plot was used to visually assess publication bias in the meta‐analytic model (Figure 2). Results from the regression for funnel plot asymmetry were nonsignificant (*z* = 0.859, *p* = .390), suggesting that the results were not biased by missing studies. However, results from the trim and fill method suggested six studies that were potentially missing from the meta‐analytic sample (i.e., due to publication bias). The re‐estimated overall correlation coefficient including the imputed studies was lower than the original estimate (overall re‐estimated correlation = .040, *SE* = 0.036, 95% CI: [−0.030, 0.109], *p* = .267).

## 4 DISCUSSION

Research assessing brain–behavior associations between hippocampal volume and memory performance in children has increased in quantity in recent years yet has also yielded mixed findings. This meta‐analysis quantitatively assessed the relation between total hippocampal volume and memory in children and adolescents across a total of 25 studies. As hypothesized, results showed a small, positive relation between total hippocampal volume and memory, such that larger volumes were related to better memory performance. Analyses assessing heterogeneity suggested a small amount of variability across study estimates. Contrary to our hypotheses, this was not explained by variations in memory task type (i.e., immediate, delayed, nonrelational, relational). Furthermore, this was not explained by variations in the age range of participants or the method used to normalize hippocampal volumes.

### 4.1 Associations between total hippocampal volume and memory

Findings show that across age and memory task types, larger total hippocampal volume is associated with better memory ability. These findings contrast those from a previous meta‐analysis, which indicated a negative relation between hippocampal volume and memory in children and adolescents (Van Petten, 2004). However, in this previous meta‐analysis, only two studies of children younger than 17 years old were included, reflecting the limited research assessing brain–behavior relations in children at the time. The current meta‐analysis offers greater clarity on this topic as it included 25 studies and 61 dependent memory outcomes.

These findings are consistent with research in healthy adult samples which shows that a larger hippocampus is more advantageous for memory performance (e.g., Maguire et al., 2000; Van Petten, 2004). As a whole, the hippocampus gains volume across development, suggesting that a larger hippocampus is likely more mature in nature and is related to superior memory ability (Canada et al., 2020; Tamnes et al., 2018). Findings of a positive association between the hippocampus and memory in childhood suggests that there may be continuity in hippocampus memory associations, such that children, adolescents, and adults exhibit a similar positive association between total volume of the hippocampus and memory ability. This consistency across development may also explain why age did not contribute to variations in study estimates.

#### 4.1.1 Impact of memory task type

Estimates across studies did not significantly vary based on memory task type. Specifically, a larger hippocampus was related to superior performance on tasks assessing immediate memory, delayed memory, nonrelational memory, and relational memory. Therefore, these findings provide support for the role of the hippocampus across memory types in childhood and adolescence. This differs from our hypotheses focused on relational memory and differs from research in adult samples, which typically highlights the hippocampus' importance for relational memory compared to nonrelational memory stimuli (Davachi, 2006). Some functional work in developmental samples show that the hippocampus may not reliably differentiate between relational and nonrelational memory in children as in adults. Specialization of the hippocampus is thought to occur throughout childhood and adolescence (Ghetti et al., 2010; Sastre et al., 2016), and similar work suggests that the hippocampus' contribution to relational memory ability throughout childhood may be nonlinear (Selmeczy et al., 2018). Therefore, it is possible that our results reflect the notion that the hippocampus is not fully specialized for relational memory in children. The results of the meta‐analysis are consistent with these developmental findings.

Tasks that are used to assess memory in developmental samples vary substantially given that children's memory abilities vastly differ throughout development. In contrast to developmental studies, tasks used in healthy adult samples are often more similar across studies. Relatedly, heterogeneity in the analyses was not explained by the hypothesized factors (i.e., age range, task type, and normalization method). This suggests that other factors contribute to the differences between study results. The current study offers an assessment of three factors, but additional dimensions of memory task type should be explored in greater detail. Therefore, although this meta‐analysis does not provide strong evidence regarding the importance of task type in understanding the association between hippocampal volume and memory, the use of more reliable and similar tasks in future research is still important and could aid in our further understanding of these associations (see Canada et al., 2021b for further discussion of this issue).

#### 4.1.2 Heterogeneity across studies

The heterogeneity analysis indicated a small degree of heterogeneity across studies. However, it was somewhat surprising that this heterogeneity was not explained by the factors investigated, which may be due to the discrepancies that exist in what tasks should be used, how age ranges should be defined, and what method should be used to normalize volumes. Although it is difficult to parse the impact of these factors given the current literature, it may become more apparent as future work adds to the number of studies that assess each age range, memory task type, and normalization method. In addition, tasks likely varied on dimensions beyond those possible to consider in this study (delay interval and relational memory component). For example, tasks may have had spatial vs. temporal components or assessed different forms of relational memory (e.g., item‐space vs. item‐time).

### 4.2 Considerations for the field

In the sections below, we discuss considerations for the field to work toward so that data can be easily shared, and findings generalized.

#### 4.2.1 Memory tasks

First, the field should aim toward the inclusion of similar memory tasks across studies. The memory tasks included in this meta‐analysis varied greatly with some studies using standardized tasks, such as the CMS or the NIH Toolbox Picture Sequence Memory Task, and others using tasks developed in house. Using similar tasks across studies would make it easier to classify memory types in order to compare across studies. In addition, there are no standard tasks to assess different memory types in child samples. Working toward utilizing similar memory tasks will require a great deal of collaboration and cooperation both within labs and across labs. This goal will also require researchers to prioritize incorporating standardized memory tasks suitable for different ages given that what is appropriate for an older child may not be appropriate for a younger child. Researchers in the field of the developmental neuroscience of memory discussed this topic during a recent conference roundtable and have been actively working toward making this a reality (Riggins, 2019). In addition, utilizing factor analysis or structural equation modeling to estimate latent measures of memory from several memory tasks would be informative as results would not rely on a single task (Canada et al., 2021b). Measured variables inherently have noise associated with them, and using a latent construct not only allows for including multiple indicators of episodic memory, but also reduces measurement error.

#### 4.2.2 Age ranges

Second, for studies combining age groups, it is helpful to report results for different age groups separately. Although this meta‐analysis suggested that differences in age range did not account for variability in study estimates, because the studies span such a large age range, it is difficult to know how findings may differ for younger compared to older children. This is especially important to consider as there are suggestions of different associations between age groups within the same studies (e.g., Canada et al., 2019; DeMaster et al., 2014; Riggins et al., 2015; Schlichting et al., 2017).

#### 4.2.3 Normalization of hippocampal volumes

Third, the field should strive to use similar methods for normalizing hippocampal volumes across studies. There was a fair amount of variability in the way that differences in head size were addressed. Our findings suggest that these differences did not contribute to variation across studies. Nonetheless, it would be useful if there was more agreement on the most appropriate method to use. The most common method used to normalize volumes was to correct for ICV/TBV using an analysis of variance approach (Raz et al., 2005). Several studies also controlled for ICV or TBV using a regression approach. The issue of normalization of volumes is complex and the answer may differ based on the age of participants.

#### 4.2.4 Reporting statistics

Fourth, we recommend that researchers fully report statistics associated with their data. Specifically, we suggest including all zero‐order correlations as they allow for comparing results across studies. Also, in many studies, results were only reported for significant findings or for patient groups. It would be useful for researchers to include statistics for all findings, including nonsignificant findings and those from typically developing control groups. In addition, reporting full descriptive statistics associated with the data is useful to understand how findings may differ for different samples of children. It is especially important that demographic variables, such as socioeconomic status, are reported to understand how these brain–behavior associations may vary across different groups.

#### 4.2.5 Assessing subregions and subfields

Associations between the hippocampus and memory may differ across subfields and subregions given that the hippocampus is a heterogeneous structure with different cell types and connectivity distributed throughout (Duvernoy, 1998; Insausti & Amaral, 2012). Assessing subunits of the hippocampus will likely provide important information regarding the relation between hippocampal volume and memory task type as subfields are thought to vary in function. Unfortunately, there are a limited number of studies (*n* = 10 for subregions and *n* = 8 for subfields) that have assessed this question in developmental samples, which prevented a robust, quantitative evaluation of the literature in its current state. More research is needed to fully assess the association between volume of each subunit and memory using meta‐analysis.

It will also be important to assess whether associations between these subunits and memory differ by age given research suggesting that these brain–behavior relations differ throughout development such that younger children exhibit one direction of effects and older children exhibit another direction of effects (see Riggins et al., 2015; Schlichting et al., 2017 for empirical demonstrations and Riggins et al., 2020 for discussion). However, the extant literature is characterized by a small number of studies often with large age ranges that may obscure age‐specific effects only apparent at particular times in development. For example, in the subfield literature, there are few studies assessing early childhood, no studies assessing middle childhood only, and one study assessing adolescence. This suggests that more research is needed to thoroughly understand the intricate relation between subregions/subfields and children's memory ability at different developmental timepoints. Fortunately, the field is growing rapidly. All studies focused on subregion and subfield volumes came out within the last 7 years given advances in technology and increased awareness of the heterogeneity of the hippocampus. In just under 10 years, sample sizes have increased substantially. This will likely produce more reliable and stable estimates and aid in our understanding of these subunits of the hippocampus and memory.

Our preliminary assessment of the literature focused on hippocampal subunits indicated that there is less variability in how hippocampal subfield and subregion volumes were normalized compared to studies assessing total hippocampal volume. All studies assessing subregions and subfields used a normalization technique and most used the same analysis of covariance technique (i.e., Raz et al., 2005) suggesting some agreement among researchers as to how to take this into account. This also suggests that researchers assessing subregion and subfield volumes in relation to memory are using similar techniques and that there is more variability among studies assessing total hippocampus.

## 5 LIMITATIONS AND FUTURE DIRECTIONS

We defined our delay using a >10‐min cut‐off. We acknowledge that others may have different definitions for what constitutes delayed memory. Our choice of this cut‐off was based on research; yet future research could assess additional cut‐offs to see if one more robustly relates to volume of the hippocampus. In addition, tasks varied on dimensions beyond delay interval and relational memory component. Future research should focus on assessing these additional delineations and classifications of memory tasks. For example, assessing spatial versus temporal memory may be appropriate given research showing that there may be anterior/posterior division with this type of memory (Poppenk et al., 2013; Ryan et al., 2010). In addition, assessing different forms of relational memory may be important (e.g., item‐space vs. item‐time; Lee et al., 2020; Giovanello et al., 2009).

Studies were omitted from the analyses if statistics were not reported, or we did not receive a response from the author. This limitation, which further underscores the importance of providing complete statistics even if they are nonsignificant, may have artificially flattened or inflated the effect. It is difficult to know if this occurred given that results from the publication bias analyses provided conflicting evidence as to whether there was bias due to missing studies. An additional limitation is that these analyses did not focus on hemispheric differences due to the limited the number of studies reporting hemispheric specificity.

Future research should also assess the impact of different tracing protocols and the use of automated software, such as FreeSurfer, to segment subfields across labs and studies. Variations in tracing protocols may make it difficult to compare findings across studies (e.g., boundaries of subfields may differ across studies). Fortunately, the field is actively working toward the harmonization of manual tracing protocols (i.e., the Hippocampal Subfields Group, 2014), which will begin to address these issues and make it easier to compare across studies. In addition to differences in manual tracing protocols, automated approaches are becoming more common to delineate subregions and subfields. However, this greater ease can be accompanied by less precision, especially if these subunits are defined using lower resolution scans (Wisse et al., 2021). In general, it will be helpful to assess how brain–behavior relations vary with regards to the segmentation method used (i.e., manual vs. automated).

## 6 CONCLUSION

Over the last decade, research examining brain–behavior relations in developmental populations has increased and yielded mixed results. The findings of the reported meta‐analyses provide clarity for the relation between total hippocampal volume and memory in children by suggesting that there is a positive association between hippocampal volume and memory that is similar across age groups and memory task types. Findings also provide an assessment of the state of the literature focused on the relation between hippocampal subfield and subregion volumes and memory. It is our hope that researchers will take the considerations discussed above into account to improve the field and make findings more generalizable across studies.

## Supporting information


**TABLE S1** Memory task classifications for each measure included in the total hippocampal volume meta‐analysis.

**TABLE S2** Studies assessing hippocampal subregion volumes and memory.

**TABLE S3** Studies assessing hippocampal subfield volumes and memory.

## Data Availability

The data that support the findings of this study are available from the corresponding author upon reasonable request.
