# Supplementary material for: A meta‐analysis of the relation between hippocampal volume and memory ability in typically developing children and adolescents
Source: Hippocampus. 2022 Mar 17;32(5):386–400. doi: 10.1002/hipo.23414 (PMC9313816; doi:10.1002/hipo.23414)
Supplement: Supplementary file 1 — TABLE S1 Memory task classifications for each measure included in the total hippocampal volume meta‐analysis. [file HIPO-32-386-s003.docx]

**Supplemental Material**

**Table S1**

*Memory task classifications for each measure included in the total hippocampal volume meta-analysis*

| Study | Memory Measure | Relational Memory | Delay |
| --- | --- | --- | --- |
| Barch et al., 2019 | NIH Toolbox Picture Sequence Memory Test | Relational | Immediate |
| Bauer et al., 2018 | Self-Derivation through Integration (Stem Facts–Open Ended) | Non-Relational | Delayed |
|  | Self-Derivation through Integration (Stem Facts–Total) | Non-Relational | Delayed |
|  | Self-Derivation through Integration (Integration Facts–Open Ended) | Relational | Delayed |
|  | Self-Derivation through Integration (Integration Facts–Total) | Relational | Delayed |
| Brunnemann et al., 2013 | RCFT (Delayed Recall) | Non-Relational | Delayed |
| Chaddock et al., 2010 | Item Memory | Non-Relational | Immediate |
|  | Relational Memory Task (d') | Relational | Immediate |
| Cooper et al., 2015 | CMS Verbal/Visual (Immediate Recall) | Non-Relational | Immediate |
|  | CMS Verbal/Visual (Delayed Recall) | Non-Relational | Delayed |
|  | Memory Component | Non-Relational | Delayed |
| DeMaster et al., 2014 | Color/Spatial Memory (Source Memory Index) | Relational | Delayed |
| Dudek et al., 2014 | CMS Stories (Immediate Recall) | Non-Relational | Immediate |
|  | RCFT (Delayed Recall) | Non-Relational | Delayed |
|  | TOMAL Visual Selective Reminding (Delayed) | Non-Relational | Delayed |
|  | TOMAL Word Selective Reminding (Delayed) | Non-Relational | Delayed |
|  | CMS Stories (Delayed Recall) | Non-Relational | Delayed |
| Fuentes et al., 2012 | TOMAL Memory for Stories (Immediate Recall) | Non-Relational | Immediate |
|  | TOMAL Word Selective Reminding (Immediate) | Non-Relational | Immediate |
|  | TOMAL Facial Memory | Non-Relational | Immediate |
|  | TOMAL Memory for Stories (Delayed Recall) | Non-Relational | Delayed |
|  | TOMAL Word Selective Reminding (Delayed) | Non-Relational | Delayed |
| Horner et al., 2012 | Item Memory | Non-Relational | Immediate |
|  | Source Memory | Relational | Immediate |
| Lambert et al., 2017 | Context Memory Accuracy | Relational | Delayed |
| Lambert et al., 2019 | Paired Associates Learning | Relational | Delayed |
| Lambert et al., 2020 | Context Tasks (d') | Relational | Delayed |
| Lee et al., 2020 | Triplet Binding Task (Item–Time) | Relational | Delayed |
|  | Triplet Binding Task (Item–Space) | Relational | Delayed |
|  | Triplet Binding Task (Item–Item) | Relational | Delayed |
| Martinos et al., 2012 | Novelty Preference (Immediate) | Non-Relational | Immediate |
|  | Novelty Preference (Delayed) | Non-Relational | Delayed |
| Ostby et al., 2012 | RCFT (30–min Recall) | Non-Relational | Delayed |
|  | RCFT (1–week Recall) | Non-Relational | Delayed |
|  | RCFT (1–week Retention) | Non-Relational | Delayed |
| Piccolo et al., 2018 | NIH Toolbox Picture Sequence Memory | Relational | Immediate |
| Raffington et al., 2019 | Item-Association Memory Task (Immediate Recall) | Relational | Immediate |
| Riggins et al., 2015 | Source Memory | Relational | Delayed |
| Riggins et al., 2018 | CMS Stories (Immediate Recall) | Non-Relational | Immediate |
|  | CMS Stories (Delayed Recall) | Non-Relational | Delayed |
|  | Temporal Order Recall | Relational | Immediate |
|  | Source Memory | Relational | Delayed |
| Dougherty & Riggins, 2013 | CMS Stories (Immediate Recall) | Non-Relational | Immediate |
|  | CMS Stories (Delayed Recall) | Non-Relational | Delayed |
|  | Source Memory | Relational | Delayed |
| Schlichting et al., 2017 | Statistical Learning | Non-Relational | Immediate |
|  | Associative Inference (inference Performance) | Relational | Immediate |
|  | Associative Inference (Direct Pair Performance) | Relational | Immediate |
| Trontel et al., 2013 | TOMAL Object Memory (Immediate Recall) | Non-Relational | Immediate |
|  | TOMAL Visual Search (Immediate Recall) | Non-Relational | Immediate |
|  | TOMAL Facial Memory (Immediate Recall) | Non-Relational | Immediate |
|  | TOMAL Visual Selective Reminding (Delayed) | Non-Relational | Delayed |
|  | TOMAL Facial Memory (Delayed Recall) | Non-Relational | Delayed |
| Willoughby et al., 2008 | CMS Stories (Immediate Recall) | Non-Relational | Immediate |
|  | CMS Stories (Delayed Recall) | Non-Relational | Delayed |
|  | CMS Word Pairs (Immediate Recall) | Relational | Immediate |
|  | CMS Word Pairs (Delayed Recall) | Relational | Delayed |
|  | RCFT (Delayed Recall) | Non-Relational | Delayed |
| Yu et al., 2017 | Visual-Auditory Learning (Immediate) | Relational | Immediate |
|  | Visual-Auditory Learning (Delayed) | Relational | Delayed |
| Yurgelun-Todd et al., 2003 | WAIS Digit Symbol (Delayed Recall) | Non-Relational | Delayed |
