# Supplementary material for: A meta‐analysis of the relation between hippocampal volume and memory ability in typically developing children and adolescents
Source: Hippocampus. 2022 Mar 17;32(5):386–400. doi: 10.1002/hipo.23414 (PMC9313816; doi:10.1002/hipo.23414)
Supplement: Supplementary file 2 — TABLE S2 Studies assessing hippocampal subregion volumes and memory. [file HIPO-32-386-s002.docx]

**Supplemental Material**

**Table S2**

*Studies assessing hippocampal subregion volumes and memory*

| **Study** | **N** | **Mean Age (yrs)** | **Age Range (yrs)** | **% Female** | **Normalization Method** | **Memory Assessment** | **Findings^c^** |
| --- | --- | --- | --- | --- | --- | --- | --- |
| Brunnemann et al., 2013 | 19 | 9.00 | 7-11 | 42 | Adjusted using covariance method | Recognition Memory (Discrimination Index (Pr)) | Larger posterior hippocampus associated with better performance |
| Daugherty et al., 2017 | 75 | 15.26 | 8-25 | 51 | Adjusted using ANCOVA | Item Memory (d') | No significant associations |
|  |  |  |  |  |  | Associative Memory (d’) | No significant associations |
| DeMaster et al., 2014 | 35 | 9.65 | 8-11 | 54 | Adjusted using ANCOVA | Color/Spatial Memory (Source Memory Index) | Larger left hippocampal tail associated with better performance. |
| DeMaster et al., 2017 | 38 | 12.30 | 8-15 | 39 | Adjusted using ANCOVA | TOMAL Visual Selective Reminding (Immediate)^b^ | No significant associations in the typically developing group |
|  |  |  |  |  |  | TOMAL Word Selective Reminding (Immediate)^b^ | No significant associations |
|  |  |  |  |  |  | TOMAL Word Selective Reminding (Delayed)^b^ | Larger anterior hippocampus associated with better performance |
| Dougherty & Riggins, 2013 | 63 | 7.20 | 5- 10 | 49 | Adjusted using ANCOVA | CMS Stories (Immediate Recall)^b^ | No significant associations |
|  |  |  |  |  |  | CMS Stories (Delayed Recall)^b^ | No significant associations |
|  |  |  |  |  |  | Source Memory | No significant associations |
| Dudek et al., 2014 | 17 | 12.30 | 11-14 | 41 | ICV/Hippocampus proportion | CMS Stories (Immediate Recall) | No significant associations |
|  |  |  |  |  |  | CMS Stories (Delayed Recall) | Larger anterior hippocampus related to better performance |
|  |  |  |  |  |  | RCFT (Delayed Recall)^b^ | No significant associations |
|  |  |  |  |  |  | TOMAL Visual Selective Reminding (Delayed)^b^ | No significant associations |
|  |  |  |  |  |  | TOMAL Word Selective Reminding (Delayed)^b^ | No significant associations |
| Lee et al., 2020 | 171 | 9.45 | 9-12 | 49 | Adjusted using ANCOVA | Triplet Binding Task (Item–Time) | Age-moderated associations between growth in head/body and performance |
|  |  |  |  |  |  | Triplet Binding Task (Item–Space) | Age-moderated associations between growth in right tail and performance |
|  |  |  |  |  |  | Triplet Binding Task (Item–Item) | Age-moderated associations between growth in head and performance |
| Riggins et al., 2015 | 22 | 4.50 | 4 | 68 | Adjusted using ANCOVA | Source Memory | No significant associations |
|  | 22 | 6.49 | 6 | 61 |  | Source Memory | Larger hippocampal head associated with better performance. |
| Riggins et al., 2018 | 176 | 6.29 | 4-8 | 55 | Adjusted using ANCOVA | Source Memory | No significant associations |
| Schlichting et al., 2017 | 41 | 11.97 | 6-17 | 51 | Adjusted using ANCOVA | Associative Inference | Smaller hippocampal head related to associated with better performance |
|  |  | 12.00 |  | 49 |  | Statistical Learning | Smaller hippocampal head related to associated with better performance |

*Note.* ^a^Indicates studies that provided partial correlations (controlled for variables other than ICV or TBV). ^b^Indicates age-adjusted memory variable. ^c^Only significant findings are reported. CMS = Children’s Memory Scale. CVLT = California Verbal Learning Test. ICV = Intracranial Volume. MST = Mnemonic Similarity Task. RCFT = Rey Complex Figure Test. TOMAL = Test of Memory and Learning.

**References**

Brunnemann, N., Kipp, K. H., Gortner, L., Meng-Hentschel, J., Papanagiotou, P., Reith, W., & Shamdeen, M. G. (2013). Alterations in the relationship between hippocampal volume and episodic memory performance in preterm children. *Developmental Neuropsychology*, 38(4), 226–235. <https://doi.org/10.1080/87565641.2013.773003>

Daugherty, A. M., Flinn, R., & Ofen, N. (2017). Hippocampal CA3-dentate gyrus volume uniquely linked to improvement in associative memory from childhood to adulthood. *NeuroImage*, *153*, 75–85.

DeMaster, D., Pathman, T., Lee, J. K., Ghetti, S., & Dem, D. (2014). Structural Development of the Hippocampus and Episodic Memory: Developmental Differences Along the Anterior/Posterior Axis. *Cerebral Cortex,* 24(11), 3036-3045. <https://doi.org/10.1093/cercor/bht160>

DeMaster, D., Johnson, C., Juranek, J., & Ewing‐Cobbs, L. (2017). Memory and the hippocampal formation following pediatric traumatic brain injury. *Brain & Behavior*, *7*(12), n/a-N.PAG.

Dougherty, L. R. & Riggins, T. (2013). The Effects of Early Experience on Brain Networks Supporting Memory during Early Childhood. *Unpublished manuscript.*

Dudek, J., Skocic, J., Sheard, E., & Rovet, J. (2014). Hippocampal Abnormalities in Youth with Alcohol-Related Neurodevelopmental Disorder. *Journal of the International Neuropsychological Society*, *20*(2), 181–191.

Lee, J. K., Fandakova, Y., Johnson, E. G., Cohen, N. J., Bunge, S. A., & Ghetti, S. (2020). Changes in anterior and posterior hippocampus differentially predict item-space, item-time, and item-item memory improvement. *Developmental Cognitive Neuroscience*, *41*. <https://doi.org/10.1016/j.dcn.2019.100741>

Riggins, T., Blankenship, S. L., Mulligan, E., Rice, K., & Redcay, E. (2015). Developmental Differences in Relations Between Episodic Memory and Hippocampal Subregion Volume During Early Childhood. *Child Development*, *86*(6), 1710–1718. <https://doi.org/10.1111/cdev.12445>

Riggins, T., Geng, F., Botdorf, M., Canada, K., Cox, L., & Hancock, G. R. (2018). Protracted hippocampal development is associated with age-related improvements in memory during early childhood. *NeuroImage*, *174*, 127–137.

Schlichting, M. L., Guarino, K. F., Schapiro, A. C., Turk-Browne, N. B., & Preston, A. R. (2017). Hippocampal structure predicts statistical learning and associative inference abilities during development. *Journal of Cognitive Neuroscience*, *29*(1), 37–51.
