# Supplementary material for: A meta‐analysis of the relation between hippocampal volume and memory ability in typically developing children and adolescents
Source: Hippocampus. 2022 Mar 17;32(5):386–400. doi: 10.1002/hipo.23414 (PMC9313816; doi:10.1002/hipo.23414)
Supplement: Supplementary file 3 — TABLE S3 Studies assessing hippocampal subfield volumes and memory. [file HIPO-32-386-s001.docx]

**Supplemental Material**

**Table S3**

*Studies assessing hippocampal subfield volumes and memory*

| **Study** | **N** | **Mean Age (yrs)** | **Age Range (yrs)** | **% Female** | **Normalization Method** | **Memory Assessment** | **Findings^c^** |
| --- | --- | --- | --- | --- | --- | --- | --- |
| Canada et al., 2019 | 68 | 6.67 | 4-8 | 54 | Adjusted using ANCOVA | MST Lure Discrimination Index | Age moderated relations between CA2-4/DG and memory performance |
|  |  |  |  |  |  | MST Item Memory | No significant associations |
| Daugherty et al., 2017 | 75 | 15.26 | 8-25 | 51 | Adjusted using ANCOVA | Item Memory (d') | No significant association |
|  |  |  |  |  |  | Associative Memory (d’) | Smaller CA3/DG related to better memory performance |
| Decker et al., 2017^b^ | 16 | 12.92 | 8-18 | NR | Adjusted using regression | Verbal Associative Memory (Immediate) | Statistics not reported |
| Keresztes et al., 2017 | 103 | NR | 6-27 | 51 | Adjusted using ANCOVA | Pattern Separation/Completion Bias | Larger DG/CA3 related to better performance |
|  |  |  |  |  |  | Source Memory | No significant associations |
|  |  |  |  |  |  | Associative Memory (Hits) | No significant associations |
|  |  |  |  |  |  | Item Memory (Hits) | Larger DG/CA3 and CA1/CA2 related to better performance |
| Keresztes et al., 2020 | 84 | 7.19 | 6-7 | 52 | Adjusted using ANCOVA | Grid Memory | Smaller left CA3/DG related to better performance |
|  |  |  |  |  |  | Lure Discrimination Index | No significant associations |
| Lee et al., 2014^a^ | 39 | 11.30 | 8-14 | 49 | Adjusted using ANCOVA | Source Hits | Larger CA3/DG related to better performance |
|  |  |  |  |  |  | Item Recognition | No significant associations |
| Riggins et al., 2018 | 153 | 6.29 | 4-8 | 55 | Adjusted using ANCOVA | Source Memory | Age-moderated relations between CA1 (head) and memory. Smaller CA1 and CA2-4/DG (body) related to better performance |
| Schlichting et al., 2017 | 41 | 11.97 | 6-17 | 51 | Adjusted using ANCOVA | Associative Inference | Age-moderated relations between CA1 and performance |
|  |  | 12.00 |  | 49 |  | Statistical Learning | Smaller CA2/3 and subiculum (head) relate to better performance |
| Tamnes et al., 2014 | 85 | 13.70 | 8-19 | 45 | None | CVLT (Immediate Recall) | No significant associations |
|  |  |  |  |  |  | CVLT (30-min Delayed Recall) | No significant associations |
|  |  |  |  |  |  | CVLT (1–week Delayed Recall) | Larger CA1 and CA2-3 related to better performance |

*Note.* ^a^Indicates studies that provided partial correlations (controlled for variables other than ICV or TBV). ^b^Indicates age-adjusted memory variable. ^c^Only significant findings are reported. CMS = Children’s Memory Scale. CVLT = California Verbal Learning Test. ICV = Intracranial Volume. MST = Mnemonic Similarity Task. NR = Not reported.

**References**

Canada, K. L., Ngo, C. T., Newcombe, N. S., Geng, F., & Riggins, T. (2019). It’s All in the Details: Relations Between Young Children’s Developing Pattern Separation Abilities and Hippocampal Subfield Volumes. *Cerebral Cortex*, *29*(8), 3427–3433. <https://doi.org/10.1093/cercor/bhy211>

Daugherty, A. M., Flinn, R., & Ofen, N. (2017). Hippocampal CA3-dentate gyrus volume uniquely linked to improvement in associative memory from childhood to adulthood. *NeuroImage*, *153*, 75–85.

Decker, A. L., Szulc, K. U., Bouffet, E., Laughlin, S., Chakravarty, M. M., Skocic, J., de Medeiros, C. B., & de Mabbott, D. J. (2017). Smaller hippocampal subfield volumes predict verbal associative memory in pediatric brain tumor survivors. *Hippocampus*, *27*(11), 1140–1154.

Keresztes, A., Bender, A. R., Bodammer, N. C., Lindenberger, U., Shing, Y. L., & Werkle-Bergner, M. (2017). Hippocampal maturity promotes memory distinctiveness in childhood and adolescence. *Proceedings of the National Academy of Sciences of the United States of America*, *114*(34), 9212–9217.

Keresztes, A., Raffington, L., Bender, A. R., Bögl, K., Heim, C., & Shing, Y. L. (2020). Hair cortisol concentrations are associated with hippocampal subregional volumes in children. *Scientific Reports*, *10*(1), 1–12.

Lee, J. K., Ekstrom, A. D., & Ghetti, S. (2014). Volume of hippocampal subfields and episodic memory in childhood and adolescence. *NeuroImage*, *94*, 162–171.

Riggins, T., Geng, F., Botdorf, M., Canada, K., Cox, L., & Hancock, G. R. (2018). Protracted hippocampal development is associated with age-related improvements in memory during early childhood. *NeuroImage*, *174*, 127–137.

Schlichting, M. L., Guarino, K. F., Schapiro, A. C., Turk-Browne, N. B., & Preston, A. R. (2017). Hippocampal structure predicts statistical learning and associative inference abilities during development. *Journal of Cognitive Neuroscience*, *29*(1), 37–51.

Tamnes, C. K., Walhovd, K. B., Engvig, A., Grydeland, H., Krogsrud, S. K., Østby, Y., Holland, D., Dale, A. M., & Fjell, A. M. (2014). Regional hippocampal volumes and development predict learning and memory. *Developmental Neuroscience*, *36*(3–4), 161–174.
